# Prevalence of Depressive Symptoms in a Predominantly Hispanic/Latinx South Texas Community in the Aftermath of the COVID-19 Pandemic

**DOI:** 10.3390/healthcare12202035

**Published:** 2024-10-14

**Authors:** Yuxia Huang, Zhiyong Hu, Ana Guerrero, Emily Brennan, Xavier F. Gonzales

**Affiliations:** 1 Department of Computer Science, Texas A&M University-Corpus Christi, 6300 Ocean Drive, Corpus Christi, TX 78412, USA; aguerrero32@islander.tamucc.edu (A.G.); ebrennan@islander.tamucc.edu (E.B.); 2 Department of Environmental Studies, University of West Florida, Pensacola, FL 32514, USA; zhu@uwf.edu; 3 Department of Life Sciences, Texas A&M University-Corpus Christi, 6300 Ocean Drive, Corpus Christi, TX 78412, USA; xavier.gonzales@tamucc.edu

**Keywords:** depression, Hispanic or Latino, South Texas

## Abstract

Objective: COVID-19 has a lasting impact on mental health, particularly within the Hispanic/Latinx communities. This paper empirically investigates the post-COVID-19 presence and severities of depression, one of the most common mental health disorders, among adults in a predominantly U.S.-born Hispanic/Latinx community in South Texas composed primarily of Mexican Americans. Methods: Multiple statistic regression models were applied to data from 515 adults in Nueces County who completed all questions in a survey from convenience sampling between June 2022 and May 2023. Depression was assessed using both standard PHQ-2 and PHQ-9 measurements. Results: Of the 515 participants, 377 (64.5%) were Hispanic, and 441 (85.6%) had a high school education or higher, reflecting the county’s demographics. About half of the participants (47%) reported mild/moderate to severe depression. The regression model estimation results reveal that female participants, those not in full-time employment, and individuals with disabilities were more likely to feel depressed after COVID-19. Middle-aged adults demonstrated greater resilience to depression compared to other age groups. Notably, non-Hispanic participants in the study reported higher levels of depression compared to their Hispanic counterparts. Additionally, COVID-19-related experiences, such as testing positive for the virus, being hospitalized, or having a history of depression before COVID-19, were associated with higher levels of reported depression. Conclusions: COVID-19 has significantly impacted the mental health of this predominantly U.S.-born Hispanic/Latinx community. These findings can assist healthcare providers and policymakers in developing targeted strategies to tailor interventions aimed at enhancing mental health well-being, reducing disparities, and fostering overall improvement within the Hispanic/Latinx community.

## 1. Introduction

Globally, the COVID-19 pandemic has led to a significant increase in mental health issues. According to the World Health Organization (WHO), during the first year of the COVID-19 pandemic, the worldwide prevalence of depression and anxiety surged by over 25% [1]. In the United States, the percentage of adults experiencing symptoms of depression or anxiety disorder rose from 36.4% in August 2020 to 41.5% in February 2021 [2]. Within the Hispanic/Latinx community, mental health emerges as a pressing concern, further exacerbated by the pandemic’s impact [3,4,5]. According to a national survey on drug use and health by the Substance Abuse and Mental Health Services Administration [6] (SAMHSA, 2019), in 2019, one in four (27%) Hispanic adults had severe mental illness —an increase of 3.7% compared to the preceding year [7]. Using a sample surveyed from across the U.S. between 13 May 2021, and 9 January 2022, another study found that one-third of Hispanic participants in the U.S. reported experiencing depressive symptoms [8].

COVID-19 has affected people’s mental health disproportionately. A substantial body of research indicates people with pre-existing mental disorders and physical health conditions are particularly at risk of developing severe symptoms of mental disorders due to COVID-19 [9]. Older patients and those suffering from chronic illness or who stayed a long duration in the hospital had higher levels of depression [10]. Furthermore, data suggest that females exhibit greater vulnerability to depression compared to males [7,11,12]. In addition, although racial/ethnic minority groups in the U.S. have been disproportionately affected by the mental health impacts of COVID-19, no clear pattern exists [3]. Most studies report that Hispanic adults are more prone to experiencing mental disorders in comparison to the non-Hispanic population [13,14]. A few studies, however, report no significant difference between Hispanic and non-Hispanic groups [3]. The prevalence of mental health also varies among Hispanic/Latinx subgroups during the pandemic. A study observes that Mexican Americans and Puerto Ricans had the highest reports of poor general mental health compared to other subgroups like Dominicans, Cuban Americans, and Central and South Americans [8].

Further, COVID-19 has a lasting impact on mental health, and its effects continue to present challenges in the post-pandemic era [15]. Studies show significantly higher depression among college students and foreign-born residents during the post-COVID-19 phase [16,17]. While numerous research studies have been conducted to understand the driving factors associated with trends in mental health during COVID-19, little research has been conducted in the post-COVID-19 phase within particular Hispanic/Latinx communities [7,18]. To comprehensively examine the intergenerational and ethnic differences in the aftermath of COVID-19 on mental health, it is crucial to consider ethnic diversity, consisting of people with different immigration statuses, socioeconomic status, and acculturation levels within the community [5]. These factors can contribute to mental health disparities along race and ethnicity lines [3]. For example, studies show second-generation Hispanic/Latinx populations had higher rates of mental health disorders compared to foreign-born Hispanic immigrants in 2018 [19]. Despite these findings, a limited number of studies have focused explicitly on mental health within predominantly Mexican American communities in the aftermath of the pandemic [3,5].

This paper fills this knowledge gap by investigating the relationship between sociodemographic variables, pre-existing mental illness, and COVID-19-related outcomes (e.g., tested positive, hospitalization, etc.) on the prevalence of depressive symptoms within Nueces County in the post-COVID-19 phase. Nueces County is a predominantly U.S.-born Hispanic/Latinx community in South Texas composed primarily of Mexican Americans. Depression is a prevalent mental health disorder characterized by persistent feelings of sadness and a loss of interest, affecting how individuals feel, think, and manage their daily activities [20,21]. The findings of this study have the potential to offer valuable insights to healthcare providers and policymakers, aiding them in devising tailored approaches to enhance mental health well-being, mitigate inequalities, and foster overall improvement within the Mexican American community.

## 2. Methods

### 2.1. Study Design and Setting

Nueces County is located along the Texas Gulf Coast. It lies approximately 150 miles north and east of the U.S. border and south of Harris and Bexar Counties, all experiencing substantial growth in the Hispanic population [22]. According to the U.S. Census [23], the county’s population in 2024 is approximately 352,988, with a growth rate of 0.2% in the past year. Roughly 90% of residents reside within the city of Corpus Christi. Hispanic/Latinx individuals represent approximately 62.9%, while the white (non-Hispanic) population accounts for 29.6%. According to the American Community Survey (ACS) data, 91.2% of Hispanic/Latinx residents in Nueces County were Mexican American [24].

The Mexican American population in Nueces County is mostly non-immigrant U.S. citizens. Based on 2020 Census data, 94.6% of Nueces County residents held U.S. citizenship, slightly above the national average. Furthermore, only 8.73% of the county’s population was born outside of the United States, lower than the national average of 13.5% [23]. Mexico emerges as the most common birthplace for foreign-born residents.

Between June 2022 and May 2023, a cross-sectional study employing a convenience survey methodology was conducted to assess the prevalence of depressive symptoms in the aftermath of the COVID-19 pandemic within Nueces County. The survey participants were selected randomly from various public events and community gathering locations, such as parks, community centers, and government buildings. To be eligible for this study, participants must be aged 18 years and older and reside in Nueces County. The survey was conducted through the web-based Qualtrics platform and in both English and Spanish. The research protocol received approval from the local institutional review board associated with the authors’ affiliations which determined that the project is exempt. This study is confidential. By completing the online survey, participants consent to participate and certify that they are 18 or older and live in Nueces County.

### 2.2. Measures

The survey consists of four sections: (1) fundamental sociodemographic characteristics, (2) COVID-19-related outcomes, (3) self-reported depression history, and (4) depression assessment.

The sociodemographic questions cover home location (including zip code, county, and home address), age, gender, ethnicity/race, income level, education level, employment status, home ownership status, health insurance, and disability. COVID-19-related outcome questions include whether the participant or any of their family members have tested positive for COVID-19, been hospitalized due to COVID-19, or experienced the loss of a family member, either within or outside their household, due to the virus. The self-reported depression history question asks participants to indicate whether they struggled with mental health before the onset of COVID-19.

Regarding the depression assessment, we employed a widely recognized Patient Health Questionaire-9 (PHQ-9) questionnaire to assess participants’ levels of depression. The PHQ-9 questionnaire consists of nine questions targeting various aspects of respondents’ well-being, including sleep, energy, appetite, and other potential depression symptoms. More specifically, these nine questions asked whether, over the last 2 weeks, they experienced the following: (1) little interest or pleasure in doing things; (2) feeling down, depressed, or hopeless; (3) trouble falling asleep, staying asleep, or sleeping too much; (4) feeling tired or having little energy; (5) poor appetite or overeating; (6) feeling bad about yourself; (7) trouble concentrating on things; (8) moving or speaking, or the opposite; and (9) thoughts that you would be better off dead or hurting yourself. The scores of 0, 1, 2, and 3 were assigned according to the response categories of “not at all”, “several days”, “more than half the days”, and “nearly every day”, respectively.

PHQ-2 and PHQ-9, two widely used types of depression screening tools, were used to gain a deeper understanding of the prevalence of depression symptoms in the aftermath of the COVID-19 pandemic. The PHQ-2, the first measure, uses the first two questions from the PHQ-9 questionnaire, which include their interest or pleasure in doing things and whether they feel down, depressed, or hopeless. Correspondingly, the PHQ-2 total score for the two questions ranges from 0 to 6. If the score is 3 or greater, a person has a positive screen, which means a major depressive disorder is likely. The individuals who screen positive should be further evaluated with the PHQ-9. The Cronbach’s alpha value among the two questions was 0. 815, and the 95% CI is 0.761–0.860, indicating a high level of internal consistency between the two questions.

The PHQ-9, the second measure, utilizes all nine questions from the PHQ-9 questionnaire. Since each question of the PHQ-9 is assigned a score ranging from 0 to 3, the cumulative PHQ-9 score ranges from 0 to 27, where higher scores indicate more severe depression. According to standard depression outcome categorizations, these scores represent three levels of depression: 0–4 (no to minimal depression), 5–14 (moderate depression), and 15–27 (severe depression). The reliability analysis results revealed a higher level of internal consistency among all nine questions (Cronbach’s alpha = 0. 924, 95% CI: 0.912–0.933).

### 2.3. Sample Size

The sample size was determined using a population proportion formula based on the following assumptions: 50% of the estimated proportion, a margin of error of 0.05, a confidence level of 95% (z-score = 1.96), an estimated non-completed rate of 12%, and a design effect of 1.4. The final sample in this study is 602.

### 2.4. Statistical Analysis

In our empirical models to investigate the impact of depression outcomes using PHQ-2 measurements, the dependent variable indicates a positive depression screen, or the presence of major depression. To estimate the binary outcome variable of having a positive depression, we applied a logistic regression model.

In addition to analyzing depression outcomes as a binary variable, we are also interested in modeling the ordered depression severity categories using the PHQ-9 measurement to measure participants’ depressive disorder further. These categories are minimal depression, moderate depression, and severe depression, reflecting the severity of depression symptoms, as described in the previous section. Following the regression with the ordered categorical variables, we applied the ordinal logistic regression to estimate the likelihood of having a major depression versus moderate or minimum depression:

(1) $$logit\;\left(P\left(Y\leq j\right)\right)=\log\frac{P\left(Y\leq j\right)}{P\left(Y>j\right)}=\beta_{j0}+\Sigma \beta_{ji}x_{i},\;j=1,2,3$$

One assumption underlying ordinal logistic regression is the proportional odds assumption or the parallel regression assumption, which means the intercepts are different for each category, but the slopes are constant across categories. Therefore, the above equation can be simplified as follows:

(2) $$logit\;\left(P\left(Y\leq j\right)\right)=\log\frac{P\left(Y\leq j\right)}{P\left(Y>j\right)}=\beta_{j0}+\Sigma \beta_{i}x_{i},\;j=1,2,3$$

The logistic regression analysis was conducted with the “glm” package using the R software, version 4.2.2. The ordinary logistic regression analysis was conducted with the “polr” package. The empirical work began by checking the effect of collinearity, then a backward selection of regressors was employed to determine the model specifications. As a result, income level is highly correlated with education level, health insurance is highly correlated with a disability, and the loss of family members due to COVID-19 is highly related to hospitalization. Therefore, these three variables, including income level, health insurance, and the loss of family members, were not included in the further analysis as reported in Section 3 below. The statistical analysis was performed at a significance level of α = 0.05.

The normal test provided by the ‘ordinal’ package was used to test the proportional odds assumption for the ordinal logistic regression. This function checks whether the effect of predictors is the same across different levels of the ordinal outcome, which is what the proportional odds assumption entails. The high *p*-values from the test (above 0.05) indicate that the proportional odds assumption holds for the predictors, meaning the model’s proportional odds assumption is valid. If the *p*-value associated with a predictor is low (below 0.05), the proportional odds assumption is violated, indicating that this effect changes across different levels of the outcome variable.

## 3. Empirical Results

A total of 602 participants participated in the survey. Of these, 515 completed all questions, resulting in a completion rate of 86%. Only the responses from these 515 participants were used for the analysis. Table 1 displays the descriptive characteristics of the participants and their depression outcomes. Overall, 280 (54.37%) were between 18 and 39 years old (young adults), 171 (31.20%) were between 40 and 59 years old (middle-aged adults), and 64 (12.43%) were 60 years older or over (senior adults). There were 393 female participants, which makes up 76.31% of the total.

Notably, the participants’ race and ethnicity distribution closely mirrored the demographic composition of the county. This is particularly evident in the representation of Hispanic respondents, who made up 332 out of 515 (64.47%), closely matching their 62.9% share of the county population. For education, about 85.63% of respondents had completed at least some college, with a substantial 242 (46.99%) having accomplished at least a bachelor’s degree.

Among the 515 participants in this study, 362 (70.29%) were homeowners, 308 (59.81%) were employed full-time, and 66 (12.92%) reported having a disability. The significant majority (82.33%) claimed that they had tested positive for COVID-19, while about one-third (32.63%) stated that a family member had been hospitalized due to COVID-19. In terms of mental health history, 215 participants (41.75%) reported experiencing depression prior to the pandemic. These findings provide valuable context for understanding the participants’ background and how these factors might relate to the impact of COVID-19 on depression outcomes.

The average PHQ-9 score among the participants is 5.58, with the scores for each category detailed in Table 1. The group that reported prior depression had the highest average PHQ-9 score (10.0), nearly four times higher than those without such concerns (2.71). Other groups with high average PHQ-9 scores include individuals with disabilities (7.95), renters (7.94), young adults (7.43), and those not employed full-time (7.0). In Table 1, bold numbers indicate the category within each group with the highest average PHQ-9 score. Young adults in particular had an average PHQ-9 score nearly twice as high as other age groups. Females and renters also show a significantly higher average of PHQ-9 scores compared to their counterparts. Regarding education, participants with a four-year degree or higher had a significantly lower average PHQ-9 score than those with less education. Interestingly, the average PHQ-9 scores for Hispanics (5.77) and non-Hispanics (5.74) were very similar. Additionally, the differences between the groups regarding COVID-19 positivity and hospitalization were not as pronounced as those observed in the sociodemographic groups. These patterns of differences and similarities motivate us to apply regression analysis to further understand the prevalence of depressive symptoms in the aftermath of the COVID-19 pandemic.

**Figure 1:**
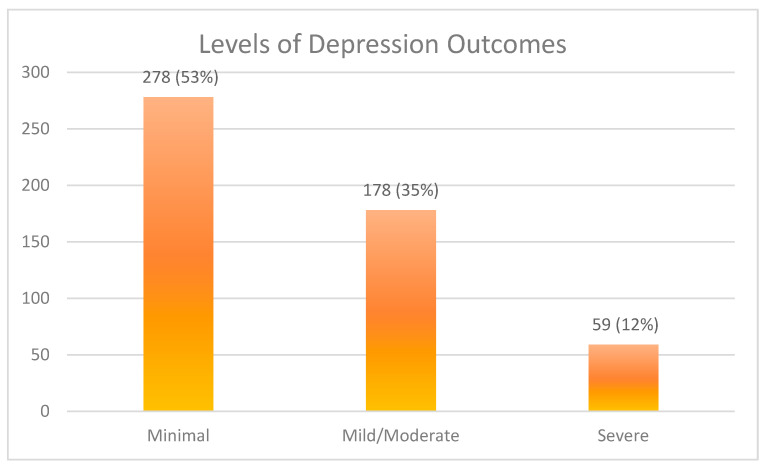
Levels of depression concerns among participants.

Further, of the 515 participants, 278 (53%) reported minimal depression, with PHQ-9 scores of less than 4. Meanwhile, 178 (35%) reported moderate depression, with PHQ-9 scores ranging from 5 to 14. Lastly, 59 participants (12%) reported severe depression, with PHQ-9 scores greater than 15. From these results, we can see that 238 participants (47%) experienced mild/moderate to severe depression concerns (Figure 1).

### 3.1. Logistic Analysis Results on Mental Health Outcome Based on PHQ-2 Measurement

Table 2 presents the results of the logistic regression analysis, including the Akaike Information Criterion (AIC) on depressive disorders based on the PHQ-2 measurement. Depression outcomes are categorized as either “no major depression” or “major depression”. Variables without estimates represent the reference groups or baselines. The results are presented as coefficients and odds ratios (ORs) alongside their corresponding 95% confidence intervals (CIs). A positive coefficient, which corresponds to an odds ratio greater than one, indicates that the odds of experiencing depression are higher for the corresponding benchmark group, holding other covariates constant. Conversely, a negative coefficient, with an odds ratio of less than one, suggests a lower likelihood of reporting depression.

The logistic regression was first conducted for each independent sociodemographic variable separately (Model 1) to examine bivariate associations between the dependent variable and each of the seven independent variables. As a result, these univariate logistic regression results indicate that all sociodemographic variables except for housing are significantly associated with depression outcomes. Middle-aged adults had lower odds of reporting major depression issues compared to young adults [OR = 0.087, 95% CI: 0.058–0.128, *p* < 0.01]. Females were more likely to report depression issues than males [OR = 1.741, 95% CI: 0.932–3.251, *p* < 0.1]. Interestingly, non-Hispanic participants had significantly higher odds of being more likely to have major depression concerns in the post-COVID-19 era compared to Hispanic participants [OR = 1.1593, 95% CI: 1.035–2.449, *p* < 0.01]. Participants with some college education were less likely to report major depression issues compared to those with less than high school [OR = 0.503, 95% CI: 0.280–0.903, *p* < 0.05]. Participants who rented their accommodations, were not employed full-time, or had a disability had higher odds of reporting major depression [OR = 1.291, 95% CI: 0.826–2.017, *p* > 0.1], [OR = 1.561, 95% CI:1.015–2.402, *p* < 0.05], and [OR = 2.429, 95% CI:1.490–3.960, *p* < 0.01], respectively, compared with their counterparts.

The seven independent sociodemographic variables were then analyzed simultaneously in a logistic regression (Model 2). Their associations with the dependent variable, namely depression outcome, remained consistent. The odds of reporting major depression issues were lower for middle-aged adults [OR = 0.578, 95% CI: 0.267–1.250, *p* > 0.1] and for those with some college education [OR = 0.454, 95% CI:0.241–0.856, *p <* 0.05] compared to their counterparts. In contrast, females [OR = 1.787, 95% CI: 0.931–3.429, *p <* 0.1], non-Hispanics [OR = 2.073, 95% CI: 1.300–3.305, *p <* 0.05], those not employed full-time [OR = 1.178, 95% CI: 0.726–1.910, *p* > 0.1], and individuals with disabilities [OR = 2.720, 95% CI: 1580–4682, *p <* 0.01] were likely to report major depression compared to their counterparts. The associations of gender, ethnicity, education, and disability with depression outcomes remained significant.

In Model 3, after including the COVID-19-related variables (COVID-19 positive and COVID-19 hospitalized), the associations between the sociodemographic variables and depression outcomes remained consistent. The COVID-19 hospitalized variable was statistically associated with depression outcome. Participants who had a family member hospitalized due to COVID-19 were more likely to report major depression [OR = 1.767, 95% CI: 0.951–3.283, *p <* 0.1] compared to those without such an experience.

Finally, in Model 4, after including the depression history variable, namely the prior COVID-19 depression concerns, the associations between all sociodemographic variables and COVID-19-related variables and depression outcomes remained consistent. Prior COVID-19 depression concerns continue to demonstrate significant associations with current depression. Participants with pre-existing depression issues were eight times more likely to report major depression [OR = 8.485, 95% CI: 3.813–18.878, *p <* 0.01] compared to those without prior depression concerns.

### 3.2. Ordinal Logistic Analysis Results on Mental Health Outcomes Using PHQ-9 Measurement as Dependent Variable

Table 3 presents the results from the ordinal logistic regression model, including the intercepts, residual deviance, AIC, and accuracy. The dependent variable is categorized into three levels of depression outcomes: no/minimal, mild/moderate, and severe, measured using PHQ-9 scores. All independent variables from Model 4 in the previous logistic regression analysis are included.

Overall, the results from the ordinal logistic regression are consistent with those from the logistic regression. Variables such as age, gender, disability, COVID-19 positive, COVID-19 hospitalized, and a history of depression were statistically significantly associated with depression outcomes. Middle-aged participants were less likely to report moderate or severe depression issues than young adults [OR = 0.522, 95% CI: 0.315–0.840, *p <* 0.001] when controlling for other factors. In contrast, females were more likely to develop a higher order of depression than males [OR = 1.363, CI: 0.976–1.923, *p* = 0.07]. Participants with a disability had a higher likelihood of experiencing a higher level of depression concerns [OR = 1.553, 95% CI: 1.039–2.313, *p* = 0.031] compared to those without disability issues.

The risk of having a higher level of depression concerns was nearly twice as high among participants who had been COVID-19 positive [OR = 1.827, 95% CI: 1.095–3.043, *p* = 0.021] compared to those who had not. Those who had been hospitalized due to COVID-19 were also more likely to develop a higher level of depression [OR = 1.635, 95% CI: 1.071–2.484, *p* = 0.022] compared to those without hospitalization experience. Participants with a history of depression issues were almost five times more likely to develop a higher level of depression [OR = 4.883, 95% CI: 3.646–6.605, *p <* 0.001] compared to those without previous depression issues. Non-Hispanic participants, homeowners, and those not employed full-time were more likely to develop a higher level of depression, with odds ratios of 1.164, 1.229, and 1.092, respectively, compared to their counterparties.

## 4. Discussion

This study examines the depression outcomes in Nueces County, a predominantly U.S.-born Hispanic/Latinx community composed overwhelmingly of residents from Mexican origin, in the aftermath of the COVID-19 pandemic. Our regression results confirm the role of sociodemographic factors, pre-existing mental illness, and COVID-19-related outcomes that impact the prevalence of depressive symptoms. These findings complement the ongoing and future surveillance efforts to determine tailored interventions for Mexican Americans in the U.S./Mexican border region and throughout the U.S. According to the U.S. 2020 Census [25], the Hispanic/Latinx population made up 18.73% of the U.S. population in 2020. A 2023 national population projection by the U.S. Census Bureau estimates that the Hispanic population will reach 111 million by 2060, meaning that over one in four Americans will be Latino [26]. Mexican Americans are the largest subgroup within the U.S. Hispanic/Latinx population, accounting for 10.7% of the total U.S. population and nearly 60% of the Hispanic population [27].

In alignment with existing literature, our regression results confirm the role of sociodemographic factors in reflecting the impact of COVID-19 on depression outcomes. A substantial body of research underscores that depression problems are more prevalent among females than males [11,28]. Our findings further support this, showing that the female population in Nueces County experienced higher levels of depression in the aftermath of the COVID-19 pandemic compared to the male population. Gender also reflects economic insecurity, which in turn affects mental health outcomes. According to 5-Year Census ACS data [29], women in Nueces County earn approximately 70% of what men earn. Furthermore, the number of female-headed households with no spouse present is approximately three times higher than that of male-headed households. Additionally, the average household size for female-headed households is larger than that of male-headed households.

Research indicates that within the Hispanic/Latinx population, older adults and young individuals are more vulnerable to experiencing mental distress [30]. This study adds to the existing literature by explicitly examining the prevalence of depression symptoms in a predominantly Hispanic/Latinx community in the post-COVID-19 phase. Our findings reveal that depression among young adults and seniors in Nueces County was less resilient in the aftermath of the pandemic compared to middle-aged adults. Income levels may contribute to this observed trend. In Nueces County, median income varies across different age groups. According to Census ACS data [29], residents aged 45 to 64 have the highest median income, exceeding $70,800, while residents under 25 and those aged 65 and over have the lowest median income, at $37,000 and $44,500, respectively.

Regarding ethnicity, our empirical results contradict earlier studies that suggested a higher risk of depressive symptoms among the Hispanic population compared to the non-Hispanic population. Due to the diverse backgrounds of the population, including variations in countries of origin and the distinct adversities faced by each group, the prevalence of mental conditions differs across these groups [17]. Other studies assessing depressive symptoms in Hispanic/Latinx communities have attributed conflicting findings to the Hispanic/Latinx community, emphasizing a need for research focused on specific subgroups [7]. In the South Texas community from which our data were sourced, Hispanic residents in Nueces County appear to have a lower risk of experiencing depression compared to their non-Hispanic counterparts. Identifying the underlying reason for this unexpected discovery is challenging, but regional culture may play a significant role.

Social isolation and loneliness are frequently cited as stressors that contribute to the development of depression. In other words, family support and a sense of belonging play significant roles in fostering positive mental health outcomes [11,31]. Research within the Hispanic community has found that the duration of time living in the U.S., rather than ethnicity alone, is a significant predictor of psychosocial well-being [32].

The Hispanic/Latinx residents of Nueces County are predominantly non-immigrant populations of Mexican origin, mainly second- and third-generation U.S. residents. Population-based surveys indicate that Hispanic/Latinx individuals within this community have, on average, resided there longer than the non-Hispanic populations [33]. The Hispanic/Latinx culture often strongly emphasizes family and community ties, with concepts of familism and collectivism providing a robust sense of support and belonging [3].

Both significant depression disorder and persistent depression affect both thoughts and the physical body. A history of mental health outcomes continues to demonstrate significant associations with current depression. According to a 2021 health data report on Nueces County [34], a higher number of white residents died by suicide in comparison to Latinos/Hispanics in Nueces County. These data underscore the critical need for targeted mental health interventions and support services, particularly among populations at greater risk, to enhance overall community health.

Our study has some limitations. First, chronic disease and physical health have been linked to higher levels of depressive symptom reporting in Mexican Americans [17]. Our study supports the significant role of pre-existing mental illness in the prevalence of depressive symptoms. In this study, a history of being diagnosed with a depressive disorder was not available. Instead, we used self-reported depression history as a proxy to estimate participants’ depressive symptom levels prior to COVID-19. Nueces County has a high prevalence of chronic disease, with an adult obesity rate of 33.2% and a diabetes rate of 13% [35]. Second, in addition to socioeconomic disparities, other factors, such as limited access to mental health facilities, might contribute to the increased vulnerability of residents to mental health issues during the post-COVID-19 phase. Our study indicates that females and those with part-time employment show higher depression. This highlights a need to examine access to mental healthcare. In Nueces County, the areas with the highest rates of poor mental health align with zip codes facing elevated chronic disease rates [34]. There is a shortage of mental health professionals across the state and the U.S., including Nueces County. The rates of psychiatrists and psychologists per 100,000 residents in Nueces are 5.8 and 20.6, respectively, which are below the Texas averages of 7.8 and 27.1, respectively [36]. The shortage of healthcare facilities and healthcare professionals, including mental health services, coupled with the lack of health insurance and other obstacles, likely influences depression outcomes. Further investigation is needed to understand these dynamics comprehensively. Third, though this study focuses on a predominantly Hispanic/Latinx South Texas community, the majority of Hispanic residents are Mexican Americans. The risk of depressive symptoms among Hispanic/Latinx individuals varies based on ethnic subgroups [7]. Thus, the results of this study are not representative or generalizable across all predominantly Hispanic/Latinx communities or across all Hispanic/Latinx subgroups. Finally, the survey was only conducted at public events, community gathering locations, and online, which may have limited participation due to individuals’ availability or access to technology.

## 5. Conclusions

This study underscores a high prevalence of depressive symptoms among adults in a predominantly Hispanic/Latinx South Texas community in the aftermath of the COVID-19 pandemic. The ongoing impact of COVID-19 is reshaping our society, and its influence on mental health is expected to persist long after the pandemic [37]. While this study allows us to assess the prevalence of depressive symptoms shortly after the end of the pandemic in a predominantly Hispanic/Latinx South Texas community, it remains uncertain whether these trends will continue during the post-pandemic recovery phase. From this standpoint, a valuable direction for future research is to extend this study to explore the long-term effects of post-COVID-19 on depression outcomes within the predominantly Hispanic/Latinx community. Given that this community predominately comprises non-immigrant Hispanic/Latinx residents, it reflects broader trends in which the Texas and U.S. populations are heading. As such, it stands as an ideal community for examining future responses.

## Figures and Tables

**Table 1:** Descriptive characteristics of the participants and their depression outcomes.

Variable	Level	Total, *n* (%)	Depression PHQ-9, M(SD)
Age	18–39 (young adults)	280 (54.37%)	**7.43 (5.28)**
	40–59 (middle-aged adults)	171 (31.20%)	3.93 (5.12)
	60+ (senior adults)	64 (12.43%)	3.30 (4.92)
Gender	Male	122 (23.69%)	3.84 (5.28)
	Female	393 (76.31%)	**6.35 (6.24)**
Ethnicity	Hispanic	332 (64.47%)	5.74 (6.01)
	Non-Hispanic	183 (33.53%)	**5.77 (6.32)**
Education	<= high school	74 (14.37%)	**6.35 (6.95)**
	Some college	199 (38.64%)	**6.83 (6.58)**
	Four-year degree or higher	242 (46.99%)	4.68 (5.22)
Housing	Owned	362 (70.29%)	4.83 (5.60)
	Rented	153 (29.71%)	**7.94 (6.71)**
Employment	Full-time	308 (59.81%)	4.92 (6.49)
	Not full-time	207 (40.19%)	**7.00 (6.77)**
Disability	No	449 (87.18%)	**7.95 (8.05)**
	Yes	66 (12.92%)	5.43 (5.72)
COVID-19 positive	No	91 (17.67%)	5.29 (6.22)
	Yes	424 (82.33%)	5.85 (6.10)
COVID-19 hospitalized	No	347 (67.38%)	5.46 (5.74)
	Yes	168 (32.62%)	**6.50 (6.95)**
Depression history	No	300 (58.25%)	2.71 (3.81)
	Yes	215 (41.75%)	**10.00 (6.20)**

Notes: (1) The numbers in bold indicate the category in each group with the highest average PHQ-9 score. (2) M(SD): mean and standard deviation of PHQ-9 cumulative score from 0 to 27. The mean of PHQ-9 is 5.75, and the standard deviation is 6.11.

**Table 2:** Estimation results of logistic model for depression outcomes using PHQ-2 measurement as dependent variable.

		Model 1	Model 2	Model 3	Model 4
Variable	Level	Odds Ratio [Coeff.] (95% CI)	Odds Ratio [Coeff.] (95% CI)	Odds Ratio [Coeff.] (95% CI)	Odds Ratio [Coeff.] (95% CI)
Age	18–39 (young adults)	-	-	-	-
	40–59 (middle-aged adults)	0.087 [−2.443] *** (0.058–0.128)	0.578 [−0.549] (0.267–1.250)	0.598 [−0.515] (0.278–1.286)	1.283 [0.249] (0.544–3.026)
	60+ (senior adults)	1.508 [0.411] (0.378–2.914)	1.034 [0.034] (0.496–2.158)	1.075 [0.073] (0.515–2.246)	1.117 [−0.111] (0.514–2.424)
Gender	Male	-	-	-	-
	Female	1.741 [0.555] * (0.932–3.251)	1.787 [0.580] * (0.931–3.429)	1.788 [0.581] * (0.926–3.452)	1.427 [0.356] (0.703–2.829)
Ethnicity	Hispanic	-	-	-	-
	Non-Hispanic	1.593 [0.465] ** (1.035–2.449)	2.073 [0.729] ** (1.300–3.305)	2.177[0.778] *** (1.348–3.517)	2.048 [0.716] ** (1.233–3.399)
Education	<= high school	-	-	-	-
	Some college	0.503 [−0.687] ** (0.280–0.903)	0.454 [−0.790] ** (0.241–0.856)	0.450 [−0.799] ** (0.237–0.855)	0.420 [−0.867] ** (0.207–0.852)
	Four-year degree or higher	0.968 [−0.032] (0.583–1.606)	1.079 [0.076] (0.628–1.854)	1.094 [0.090] (0.633–1.887)	1.277 [0.244] (0.483–1.411)
Housing	Owned	-	-	-	-
	Rented	1.291 [0.256] (0.826–2.017)	0.979 [−0.022] (0.594–1.612)	1.009 [0.009] (0.613–1.660)	0.826 [−0.192] (0.483–1.411)
Employment	Full-time	-	-	-	-
	Not full-time	1.561 [0.445] ** (1.015–2.402)	1.178 [0.164] (0.726–1.910)	1.142 [0.132] (0.702–1.856)	1.114 [0.107] (0.664–1.869)
Disability	No	-	-	-	-
	Yes	2.429 [0.888] *** (1.490–3.960)	2.720 [1.001] *** (1.580–4.682)	2.558 [0.939] *** (1.481–4.414)	2.761 [1.015] ** (1.527–4.994)
COVID-19 positive	No	-	-	-	-
	Yes			1.545 [0.436] (0.721–3.314)	1.427 [0.356] (0.628–3.246)
COVID-19 hospitalized	No			–	–
	Yes			1.767 [0.570] * (0.951–3.283)	1.794 [0.584] * (0.917–3.507)
Depression history	No			–	–
	Yes				8.485 [2.138] *** (3.813–18.878)
AIC			297.37	298.37	253.17

Notes: * *p* < 0.1, **p < 0.05, *** *p* < 0.01. OR = odds ratio. CI = confidence interval. AIC = Akaike Information Criterion. Model 1: bivariate associations between the dependent variable and each of the seven independent variables. Models 2–4: multivariate logistic regressions; Model 2: adjusted for age, gender, ethnicity, education, housing, employment, and disability; Model 3: additional adjusted for COVID-19 positive and COVID-19 hospitalized; Model 4: additional adjusted for depression history.

**Table 3:** Estimation results of ordinal logistic regression for depression outcomes from PHQ-9 measurement.

Variable	Level	Coeff.	Std Err	*p*-Value	Odds Ratio (95% CI)
Age	18–39 (young adults)	–	-	-	-
	40–59 (middle-aged adults)	−0.650	0.259	<0.001	0.522(0.315–0.840) ***
	60+ (senior adults)	0.072	0.211	0.733	1.075 (0.708–1.624)
Gender	Male	-	-	-	-
	Female	0.310	0.173	0.073	1.363 (0.976–1.923) *
Ethnicity	Hispanic	-	-	-	-
	Non-Hispanic	0.152	0.150	0.308	1.164 (0.868–1.561)
Education	<= high school	-	-	-	-
	Some college	−0.273	0.212	0.198	0.761 (0.504–1.167)
	Four-year degree or higher	−0.183	0.172	0.288	0.833 (0.594–1.167)
Housing	Owned	-	-	-	-
	Rented	0.206	0.153	0.178	1.229 (0.909–1.656)
Employment	Full-time	-	-	-	-
	Not full-time	0.088	0.148	0.553	1.092 (0.817–1.459)
Disability	No	-	-	-	-
	Yes	0.440	0.204	0.031	1.553 (1.039–2.313) **
COVID-19 positive	No	-	-	-	-
	Yes	0.600	0.260	0.021	1.824 (1.095–3.043) **
COVID-19 hospitalized	No	-	-	-	-
	Yes	0.491	0.215	0.022	1.635 (1.071–2.484) **
Depression history	No	-	-	-	-
	Yes	1.585	0.151	<0.001	4.883 (3.646–6.605) ***
Intercepts					
	Minimal depression|mild/moderate depression	0.026	0.209	0.901	
	Mild/moderate depression|severe depression	2.626	0.244	<0.001	
Residual deviance	768.09				
AIC	796.10				
Accuracy	0.68				

Notes: * *p <* 0.1, **p < 0.05, *** *p <* 0.01. OR = odds ratio. CI = confidence interval. AIC = Akaike Information Criterion.

## Data Availability

The data are not publicly available due to privacy or ethical restrictions.
